# The lived experience of co-production: Reflective accounts from the InCLUDE project

**DOI:** 10.1186/s40900-024-00639-2

**Published:** 2024-10-14

**Authors:** Shayma Izzidien, Rachael Stemp, Sakab Akram, Sabbir Ahmed, Alay Rangel-Cristales, Karen Irvine, Shivani Sharma, Nick Midgley

**Affiliations:** 1 Anna Freud, London, UK; 2 School of Life and Medical Sciences, University of Hertfordshire, Hatfield, UK; 3 College of Business and Social Sciences, Aston University, Birmingham, UK; 4 Department of Clinical, Educational and Health Psychology, UCL, London, UK

**Keywords:** Co-production, Patient and public involvement, Participatory research, Children’s social care, Reflection, Underserved, Diaries, Ethnicity, Foster care, Kinship care

## Abstract

**Background:**

The value of co-produced research in health and social care is increasingly recognised, but accounts into the processes and individual experiences of co-producing research are lacking. This paper describes the personal journeys of four researchers (two experts by foster caring experience and two experts by profession) throughout the life course of a co-produced research project exploring the barriers and facilitators to inclusive research in foster caring, the InCLUDE project.

**Methods:**

Each researcher kept a diary throughout the InCLUDE project of their personal reflections, questions, and learning. These were synthesised and reviewed by the researchers and two colleagues external to the team, and key themes were extracted.

**Results:**

Narratives constructed from the diaries are presented in relation to distinct aspects of the co-production journey, alongside illustrative quotes. These aspects include: motivations for starting the project; making sense of the project; defining roles and responsibilities; challenges; and reflections on acquired knowledge and skills. From these insights, the researchers present recommendations for others endeavouring to engage in co-produced research. These include: recognising vulnerabilities and challenges during the early stages of a project; creating safe spaces; seeing the value of diversity; harnessing individual strengths; establishing a strong routine; and ensuring equal voice.

**Conclusions:**

This paper presents a novel perspective on the realities of co-produced research by documenting the lived experiences of researchers within the context of foster care research. It highlights the importance of both measurable, tangible project outcomes, and the personal and skills growth of team members. The consistent use of diaries is encouraged as a valuable practice to capture learning, progress and achievements throughout the co-production process.

**Supplementary Information:**

The online version contains supplementary material available at 10.1186/s40900-024-00639-2.

## Abstract (plain language summary)

Four researchers (two experts by foster caring experience and two experts by profession) reflect on their journey of being part of the InCLUDE project. InCLUDE looked at how to make research with foster and kinship carers more inclusive. Each researcher kept a diary throughout the project, to help them capture their learning, thoughts and feelings. In this paper, the diaries are collated together into themes, with illustrative quotes. These themes include motivations to get involved in the project, settling into the role, challenges, personal development, and new knowledge. The researchers then share their key learning which can help others who are hoping to co-produce a research project. This includes the importance of talking about feeling vulnerable, creating safe spaces, working to your individual strengths, setting up a strong routine, and making sure that everyone has an equal voice and role on the project. The researchers also discuss the benefits of using diaries as a way to capture learning, experiences and progress, and encourage other research teams to do the same.

**Supplementary Information:**

The online version contains supplementary material available at 10.1186/s40900-024-00639-2.

## Introduction

The needs and benefits of co-production in health and social care research are well established, yet there remains a knowledge gap in the understanding of the process and lived experience of individuals engaged in co-production [1–3]. In this paper, a diverse team of four researchers (two experts by foster caring experience and two experts by profession) summarise their learning throughout a co-produced research project. The paper is based on experiences within the InCLUDE project, which assessed the barriers and facilitators to inclusivity within foster care services and research as part of the Reflective Fostering Study. By sharing reflective accounts, curated from nine months of diary entries and memos, the researchers aim to illustrate the personal challenges, opportunities and learning from the co-production experience, as well as to detail the practical approach taken.

## Co-production within social care research

Within health and social care, co-production is increasingly recognised as a central, necessary tenet of research [1, 2], with research bodies and funders increasingly requiring public involvement to be built into project design [2, 4]. Although there is no unified definition of co-production [5], there is consensus that it necessitates collaboration between research academics and members of the public who share power and responsibility for the generation of new knowledge [6, 7]. As such, co-production breaks down traditional notions of power within academia, allowing those typically marginalised from research to collaboratively bring their expertise to share decision-making throughout a research project. As such, relationships, trust, and equality are all central to good co-production [8].

Over the last decade, there has been growing consensus around the benefits of co-production for organisations, academia, communities, and individuals [9]. Co-production can lead to both higher quality research, and increased capabilities and transformed values of all those involved [10]. For research, co-production generates higher quality outputs, by increasing the applicability and relevance of research [11, 12], and increasing research efficiency by reducing costs and increasing recruitment and retention [13]. Co-production also encourages equitable and inclusive research, by bringing multiple voices into research contexts from the start. It emphasises the importance of individual differences [14] by encouraging individuals to bring their unique talents and strengths [12, 15]. This includes those who are traditionally marginalised or underserved within research spaces, to ensure that research is reflecting the needs of the communities it serves [8, 12, 16].

Despite the clear importance and value of co-production, literature into the processes of co-production is lacking [2, 3], either through formal evaluation, reporting of outcomes, or accounts of the process within co-produced projects [17]. Instead, most existing literature focusses on reporting co-production methodologies and tools or describing how co-production contributed to service improvements [2]. Therefore, several voices within co-production literature have called for researchers to share their practical experiences of co-production [2, 3].

This paper aims to address this gap by documenting the practical and personal journeys of four researchers within a co-produced project (the InCLUDE project). Through sharing reflective accounts of their co-production journeys, this paper captures both intended and unintended learning from co-production to demonstrate the importance of both the journey and destination of co-produced projects [17]. It will also illustrate co-production within the context of foster care and in collaboration with foster carers, which is currently lacking in published literature.

The following aims form an overarching framework for the paper:

1. To report on the practical journey of the team working on the InCLUDE study—a co-produced project with foster carers.
2. To provide reflective accounts of the experience of the researchers involved in the study.
3. To explore the role of personal and professional development as an outcome of co-production, rather than focussing solely on project outputs.

## The InCLUDE project

The InCLUDE project (Increasing Collaboration and Learning with Underserved communities for Diversity and Equity) aimed to put inclusivity and accessibility at the heart of research into foster care by exploring the experiences and needs of carers from underserved communities. The project ran from April 2022 to July 2023 as part of the Reflective Fostering Study (RFS). This is a National Institute for Health and Care Research (NIHR) funded randomised control trial evaluating the clinical and cost-effectiveness of a support programme for foster and kinship foster carers, the Reflective Fostering Programme [18]. The RFS was designed to evaluate whether the Programme makes a difference to carer well-being and stress, the carer-child relationship and placement stability. The clinical trial set out to be representative in its recruitment so that the participants reflect the fostering population. This was to ensure that the results benefit the wider population and are not limited to certain groups. Such an approach leads to higher quality evidence and more credible, applicable research [19]. Demographic analysis during the pilot phase of the study highlighted that male carers, kinship foster carers and carers with South Asian heritage were less likely to participate than expected based on national and local proportions of carers. If these groups were poorly represented in the study, we could not be confident that the findings would be truly applicable to the communities for whom we were doing the research. To explore this further, and to try and make necessary changes for the main trial, the team applied for further funding from the NIHR to examine and address the barriers to participation for these groups. Through a model underpinned by the principles of participatory action research [20, 21], InCLUDE sought to elevate the voices of those within underserved communities and iteratively change the recruitment process within the Reflective Fostering Study, to improve recruitment of those who had been identified as under-represented during the pilot phase of the study (male, South Asian and kinship foster carers), and to more broadly understand the barriers and facilitators to inclusive research in foster care.

## Taking a co-production approach

We used co-production as a methodology to help overcome a history of poor representation within social care and foster care research and to achieve the goals of equality, diversity and inclusion (EDI) within research. We recognised that the best way to truly understand the experiences of the groups underserved in our research and how to meet their needs was to be led by them. By collaborating with individuals who have lived experience, we hoped to be able to better identify and address the barriers to research involvement and construct more inclusive recruitment strategies.

For us, co-production involved the active involvement, shared decision making and mutual learning between all team members, with the aim of co-producing knowledge relevant to the aims of the project. This meant that power and decisions were shared equally between team members regardless of their previous experience, and that everyone was invited to draw on their individual perspectives and strengths to benefit the overall success of the project. This often meant taking different roles in accordance with our skill-set and personal development needs. The diversity of the team, in terms of gender, ethnicity, beliefs and area of expertise enriched the project with a broad range of experience and insights.

The InCLUDE project consisted of four researchers, two with primarily research experience (RS and SI) and two with primarily foster carer experience (SAk and SAh). It was overseen by the chief investigator (NM) and the trial manager of RFS (KI), plus an academic who has expertise in addressing health inequalities and leading research related to diversity and inclusion (SS). Among the four people in the core team, Rachael is a researcher with a background in psychology and who also trained as a social worker. She is female and of white heritage. Sabbir is a foster carer with 13 years’ experience and is currently caring for four birth children and one foster child with complex needs. Alongside his wife, he has fostered 20 children in total. He is a Muslim male of South Asian heritage. Sakab is a foster carer with eight years’ experience and is currently caring for three birth and two foster children. Alongside his wife, he has fostered about 15 children in total. He is also a social worker and has been working in fostering for 12 years. He is male and of South Asian heritage. Shayma is a researcher with a background in education and social care. She is female and of mixed heritage. For all team members, this was their first time working on a research project which was co-produced throughout, although each had some experience of either participating in or conducting research with some participant involvement. The project also established an advisory group 12 of carers and care professionals, who self-identified as having expertise in the needs of at least one underserved community in fostering (the InCLUDE-ME group).

All aspects of the InCLUDE project were co-produced. Activities included: focus groups with male carers, kinship carers and/or minoritised ethnic carers to understand the barriers they faced in participating in fostering services and research; a scoping review exploring the representation of different foster carer demographics within empirical research studies [22]; a national survey, entitled Share Your Voice, about the barriers and facilitators to research participation in children’s social care; and development of a toolkit on building inclusivity into fostering services [23].

## Generating reflective accounts

### Keeping reflective diaries

Between August 2022 and May 2023, the four researchers in the core team kept a weekly electronic reflective diary of their experiences and learning from the InCLUDE project. The diaries were designed to record personal learning, questions for the team and outstanding tasks. The team devised five questions which they felt would encourage self-reflection, document learning and highlight issues for problem solving: “How are you feeling this week?”, “What have you learnt?”, “What challenges are you facing”, “Any action points” and “Anything else”. The diaries were personal, and each researcher could choose what they shared and when, and how often they completed entries.

Outside the practicalities of documenting action points and questions, the team chose to keep diaries of their co-production for several reasons. Firstly, reflective diaries help contribute to research rigour by facilitating tracking of observations, reflections, and problem-solving approaches [24] and allowing researchers to track decision-making. This felt especially necessary within this co-production project, given that co-production is intended to be an emergent approach to research [25]. Secondly, research diaries have been identified as helpful tools for enabling new researchers to make sense of research processes [26], capture new learning, and navigate the “murky waters of qualitative research” [27]. For the two experts by foster caring experience researchers, this was their first-time conducting research, so tracking learning and identifying development needs was a priority.

Thirdly, writing diaries gave the researchers a space in which to reflect on power dynamics, trust, and inclusivity within the project in a personal and safe way [28] and therefore track how closely their work was mirroring these core values of co-production [17], enabling them to address any issues in a collaborative and timely way. The diaries were a direct response to calls in the literature for researchers to record their activities, skill development and outcomes, and for more reflective accounts of the processes of co-production [2, 3].

It is worth noting that it was not the intention of the authors to share the diaries within a research paper, and the diaries were not originally kept as a form of methodology. They were initially designed as a personal and team reflection tool, for the reasons outlined above. However, as the team spent time learning about co-production research, it became clear that there is a real need for published, authentic accounts which reflect the practicalities of co-produced research, as well as challenges and reflections. Therefore, the team reviewed the diaries and identified that they might not just be helpful in supporting their own reflections within the project, but may also support others to understand the coproduction journey, especially those newer to co-production, as this team had been. At this point, the decision was made to collate and tidy the diaries into individual reflective accounts, for the purpose of sharing with others for analysis. Although we did not begin with a formal theory of reflective practice, we saw our approach as broadly in keeping with the model of reflective practice as ‘learning through and from experience towards gaining new insights of self and practice’ [29], building on Schon’s model of ‘reflection-in-action’ [30].

### Collating into reflective accounts and identifying common themes

At the end of the project, each researcher collated their diary entries into a reflective account of their journey within the InCLUDE project. The decision was made to curate entries into a reflective account so that researchers could remove any reflections they wished to keep confidential and remove any non-reflective information such as to-do lists. Each of the four team members shared their reflective accounts with each other, as well as two additional researchers: one advisor to the project who had attended most weekly meetings but had not themselves been involved in the project activities and had not kept a diary (KI), and one researcher who was familiar with the team and worked with the team in other capacities, but had not been involved in the InCLUDE project at all (AR-C). The decision was made to bring in two additional researchers to help bring an additional perspective to the diaries, and to ensure that the findings presented in this paper would be relevant and accessible to those not part of the core project team. All six researchers independently read all the reflective accounts and identified common themes, and similarities and differences. A specific framework for identifying themes was not used; however, the process loosely drew on the process of thematic analysis in that each of the six researchers familiarised themselves with all four diaries and noted down any initial thoughts for potential themes, and then went through the transcripts identifying which quotes reflected which them [31]. All six researchers then came together and discussed the themes and narratives across the accounts which they had identified. Together, they discussed which themes would be most relevant and helpful for other researchers, whilst respecting the privacy of each collaborator. They also decided to present the themes in a broadly chronological order, rather than in order of prevalence, to help capture the journey of each of the researchers as clearly as possible. The themes selected through this discussion are presented below.

## The co-production journey

In this section, we present a reflective narrative of the coproduction journey within the InCLUDE project. Drawing on the reflective accounts, generated by the four members of the core research team, the journey is presented as six themes: motivations and background; finding our feet; settling into the role and the project; challenges; reflections on learning; and personal development. The themes are arranged to tell a story that loosely maps the journey through the project. The team’s experiences of challenges, learning and personal development occurred throughout the journey but are presented in separate categories for simplicity, but this is not to say the learning was linear. We end with a section called “Reflecting on others’ diaries”, where researchers explore the value of the process of sharing and analysing the reflective accounts themselves. We considered this an appropriate way to end our co-production journey.

## Theme one: Motivations and background

In this theme we considered aspects of our diaries that shed light on how our decision to be part of InCLUDE was a consequence of our ‘motivations and background’. We saw this as the starting point of our co-production journey.

The InCLUDE team were all motivated to see positive change within children’s social care research. The diaries highlight how each person’s motivations to engage in the project were influenced by the intersectionality of their multiple identities, their social and professional experiences of the world, and their personal interests. For Sabbir it was the lived experience of discrimination that underlaid his motivation to make a change.

I faced obstacles [in my fostering role] due to my ethnicity, culture, and religion. I experienced Local Authorities to be insensitive towards ethnic groups. This causes barriers for integration, diversity, and inclusion. Due to this negative experience, I feel passionately about equality, integration, and diversity. *Sabbir*

The combination of Sakab’s life experiences, initially as a supervising social worker and later a foster carer, gave him a passion to contribute to advancement of fostering services, which he hoped this project would help him work towards.

I worked in various roles and capacities within fostering services, these included work as supervising social worker, panel member…and most recently and significantly a foster carer. I strongly believe that a range of roles and valuable experience that I gained over the years had helped me develop a unique and balanced perspective when it comes to fostering related matters. *Sakab*

Rachael was interested in learning about fostering from people who had different experiences to her own. Shayma was involved in the Reflective Fostering Study and wanted the project to improve the representation of marginalised participants in the research. Rachael and Shayma came to InCLUDE having previously consulted and collaborated with adults and young people as part of their research practice. They both recognised that these past experiences are usually classed as low-level forms of participation. InCLUDE aimed to be a co-produced project, with all team members involved in the construction and delivery of the work stream.

This project appealed to my own interest in striving for diversity and equity in research practice, and ensuring participants were inclusive of marginalised people. *Shayma*

The InCLUDE project is the first time I have worked on an entirely co-produced project. I had worked in collaboration with adults and children in research before, but on reflection those projects had been more hands-off: I would ask people for advice on methodology and findings but had already set the research agenda and outputs with other colleagues. *Rachael*

Although Rachael had some experience of social work, she and Shayma came to the project primarily as academic researchers. Sabbir and Sakab brought their experiences of being foster carers and service users. Additionally, Sakab brought his previous experience as a supervising social worker.

## Theme two: Finding our feet

The next part of the journey outlined the feelings and emotions of the team as we tried to ‘find our feet’ and understand the project. This section also included the groundwork for setting up meetings and conducting the work.

The early weeks of the project were characterised by everyone trying to make sense of the project and their roles in the team. The diaries illustrate the uncertainly and apprehension during the beginning stages, with everyone working outside their usual professional capacities and feeling outside their comfort zones.

From the start, I wanted to get our co-researchers involved in a genuine and authentic way. It took time for us all to settle in, to work out how we can work together to achieve the aims of InCLUDE. *Shayma*

Sabbir and Sakab, in-particular, reported feeling overwhelmed and out of their depth. They took considerable time to understand the significance of InCLUDE within the wider study of the large-scale clinical trial. As the project progressed, they came to realise that research expertise is not exclusively academic, and that their experience as carers made them experts. Their knowledge of ‘the psychology’ of carers, the motivations underpinning fostering and the daily challenges faced by carers helped drive the project and improved the project’s engagement with carers.

I was very surprised to feel overwhelmed and out of my depth at such an early stage…the field of research was completely new and unknown territory for me…My lack of research related experience meant that initially I was somewhat naïve about the importance of my role and about the significance of research, particularly in terms of the structure, process and robust nature of high-quality research. *Sakab*

When I first joined the InCLUDE project, I did not really understand how I would make any difference, especially when liaising with academics so felt a bit side-lined, however the team always made me feel that my input was integral thus made me feel comfortable and settled into the team very quickly. *Sabbir*

The unease and uncertainty that characterised the early stages of the project were common to all team members. This vulnerability was not always shown openly, especially by the academic researchers. Through the process of reading each other’s diaries at the end of the study, Sabbir and Sakab were comforted to find out that Rachael and Shayma also held feelings of uncertainty and apprehension.

It was humbling to learn that researchers are human after all and have feelings and that they can think in a similar way to co researchers. *Sabbir*

I was surprised at the reflections of my senior colleagues who admitted finding the initial stages of their journey quite challenging. This demonstrated to me that even the most skilled researchers face challenges too, which is encouraging in itself because it gives me hope that I can also go on to excel in my own career. *Sakab*

We decided to hold InCLUDE meetings once a week which were relaxed and informal. We took turns to chair and take minutes, although the agenda was drawn up by one of the academic researchers. Alongside project work, we spent time talking about current social issues, relating them to our own experiences, and discussing how they linked to the work of InCLUDE. Most meetings were online, with some face-to-face. Face-to-face meetings were important, especially in the early days, and helped the expert-by-experience researchers feel better connected and gave them the opportunity to meet the wider Reflective Fostering team.

InCLUDE meetings were more relaxed and informal. We spent time chatting, expressing thoughts about wider issues and talking about our experience. *Shayma*

[The] research programme commenced post-Covid, this caused a little discomfort as I did not get the opportunity to meet everyone face- to- face. However, after everyone met face-to-face, I felt a lot better about the study and how I was making a difference. *Sabbir*

Reflections from all researchers demonstrate the importance of taking the time to build a safe place for everyone to feel comfortable, and this helped the team gain confidence in themselves and the project.

It’s about creating a safe space where everyone feels listened to and not intimidated by the “research” experience, so that people can share themselves and their experiences, because this is where the best ideas and suggestions for change come from. And it’s about learning about research methods together, finding new and creative ways of working, so that you can “do” research well. *Rachael*

The accounts make multiple references to the team’s positive experiences of support; this was especially important for Sabbir and Sakab during this early phase of the project and when they encountered difficulties. Sakab found that the solution to feeling overwhelmed was to request support, keep asking questions and seek clarifications. They were both surprised by what they described as the humble nature of academic researchers, being friendly and accessible.

The InCLUDE team is very encouraging, there is a lot of support, warmth, reassurance and good vibes from everyone within the team. They have respected my needs as well as my views and opinions, which in turn has boosted my confidence and allowed me to apply myself and make contributions. *Sakab*

## Theme three: Settling into the role and the project

The third part of our journey outlined the experience of team members ‘settling into the role and the project,’ highlighting how an individual’s strengths can be harnessed and skills developed as part of being part of a co-produced project.

As the project progressed everyone grew in confidence and gained autonomy in their roles. We got a better sense of people’s personalities and strengths; and the role of taking on certain tasks in the project became more organic and based on interest.

It took us time as a team to work out everyone’s strengths and to assign tasks accordingly, but I found the most effective thing for this was asking questions and constantly checking in to make sure everyone was learning from what they were working on. I was really surprised that some tasks I find dull (e.g., reading and cleaning transcripts) others found enjoyable. *Rachael*

I did not look forward to reading journals and to extract information from them. This would be something new for me and be a challenge. For me it worked out well as this was done by my colleagues. *Sabbir*

The team diversity in terms of gender, ethnicity, beliefs, and experiences was one of the main strengths of this project. This enabled us to engage with a broad audience in an authentic way. For example, Rachael and Sakab both had experience of being professionals within the social care system but had different experiences and insights and could appreciate diverse perspectives from both professionals and carers. Similarly, data collection for the Share Your Voice survey included Sabbir and Sakab speaking to carers who preferred to give their views over the phone rather than fill in an online form. As carers themselves, they were able to empathise with the interviewees and ask appropriate follow up questions.

As male researchers, Sabbir and Sakab facilitated the focus groups with male carers. After the session, the participants commented on how this set up enabled them to express their thoughts without censorship. They also took on the role of both research facilitator and participant in the discussions. To his own surprise, Sakab felt comfortable to share his own personal story with the group, which helped further in establishing rapport with other members. In another session with minority ethnic carers, facilitated by Shayma and Sabbir, participants appreciated the presence of minority ethnic researchers. One participant also preferred the presence of an academic researcher, while another enjoyed the presence of an expert-by-experience researcher, highlighting the strength of offering diversity in the facilitation of research.

During focus group discussions, I shared personal experiences I did not imagine I would be talking about. On reflection, I think it was helpful for me and helpful in prompting and allowing other members of the group to open up. This made me think that perhaps if people gave research a try, they might find that talking about their experiences is beneficial for others as well as themselves. *Sakab*

Listening to the recordings of the male carer groups, I realised that if we had female researchers in the session, the group never would have shared with the same honesty or vulnerability that they did. This highlighted to me the importance of diversity within teams, so that people can see themselves reflected within research. *Rachael*

In his account, Sabbir speaks about the importance of building rapport both within the team and through our work with underserved groups. His own preference for face-to-face meetings also made him aware of the importance of rapport during online sessions, and the barriers created through the absence of physical interactions. His personality lent itself to taking on the role of facilitating the ice-breaker sessions, which he carried out with humour and good will.

I noticed that if I have a good rapport in a meeting setting, I am more inclusive and will participate more, hence I was appointed to do ice-breaker sessions when opening meetings. For my first meeting I researched some ice-breaker sessions and thought of some interesting short ice-breaker sessions that I have been involved in previously and adopted those ideas and put them into action. *Sabbir*

Sakab highlighted the challenges people can face in processing information, and this helped us in the design and execution of our work, both as a team and when conducting focus groups with carers.

I understood that clarity and structure would be key in organising and planning the group session. I know the personal difficulties I have in following discussions and processing information. Hence, I strongly felt that clarity and good communication would allow people of all abilities to be included by enabling them to understand the aims of session. *Sakab*

Lack of knowledge or experience was not used as a reason to restrict involvement in tasks. Despite no knowledge of kinship care, Sakab co-ordinated the social media work for Kinship Care Week. He reflected on how he learned about differences between kinship care and mainstream foster care as a result and to use the experience in a positive way.

I had to admit that I was not too familiar with the plight of kinship carers and had no prior experience of working with them During the process of putting this material together, I was able to get the gist of the challenges kinship carers face. Therefore, I would urge researchers not to be put off by lack of direct experience when undertaking research tasks, there are always ways and means to access information and still make a meaningful contribution. *Sakab*

## Theme four: Challenges

The challenges associated with co-production were an important part of the journey. This was evident from the reflective accounts which highlighted that the team faced many challenges to overcome, both personally, in terms of skills development, and in terms of working within a new context. We therefore felt it was important to document these challenges individually under their own theme, while recognising that they took place throughout the project.

### Managing different roles

The experts by foster caring experience researchers engaged with InCLUDE while simultaneously managing their roles as foster carers. Shayma and Rachael both commented on the challenges of fostering faced by Sabbir and Sakab, and how their life experiences were different to researchers.

I was struck by the challenging nature of fostering as expressed by our expert-by-experience researchers, and I really admire them for joining and using InCLUDE as a vehicle for positive social change. *Shayma*

At the start of the project the aim was to involve the experts by foster caring experience researchers in all aspects of the workstream. Sabbir and Sakab only worked one day per week, so their time was limited. It also transpired that they did not want to be involved in all aspects such as the literature review. We discovered that co-production needed to be done in an equitable rather than equal way, meaning everyone did not need to be involved in the same tasks. Sabbir and Sakab did not always attend strategic meetings, and this could be interpreted as a disruption to the power balance. However, we found the most important element was ensuring they were involved in a genuine way.

Our co-researchers did not attend all meetings, including strategic meetings with senior team members, and I question if this is a limitation of our co-production efforts. Ultimately, I think it was about everyone bringing their own experiences and feeling like an equal team member; and I hope we achieved this. *Shayma*

### Making research more accessible

InCLUDE was a sub-study of the large-scale Reflective Fostering Study, which made it more complicated to understand from the perspective of our experts by foster caring experience researchers. They also commented that the academics used acronyms and abbreviations that did not make sense, a point picked up in the diaries of all four researchers. Sabir and Sakab found that taking their time, asking questions, seeking clarifications, and realising that they did not need to understand everything all at once, helped them navigate the study. The academic researchers became more aware of the need to explain in more detail, repeat aspect of the study and be mindful of their use of academic jargon.

Despite the personal progress I was making, there were still times where I would find it difficult to get my teeth firmly into the project, I believe this was because there are so many moving parts and a lot of academic jargon. However, with the fantastic support on offer from knowledgeable and experienced colleagues, I was determined to overcome these challenges. *Sakab*

The researchers used abbreviations that was normal to them, but to me it made no sense. *Sabbir*

When you are part of a research study it is easy to slip into language that is not accessible and full of jargon. Many terms that I had been alien to me when I started at Reflective Fostering—kinship, underserved, PPI, NIHR—I was now using without explaining. *Shayma*

### Navigating the complexities between research and PPI

Shayma and Rachael had numerous conversations about the differences between co-production, Patient and Public Involvement (PPI) and research, and the overlap between these. This caused confusion in trying to work out the best approach. In the end, they worked to ensure that the same rigour and ethics were applied to co-production as to research.

Starting out I wasn’t clear about what my role would look like and how to balance the PPI focus of the project with the research nature of many of the tasks. This is something that we have grappled with throughout InCLUDE, how to balance the rigour and sometimes prescriptive nature of research with the more relational and less formal nature of PPI. *Rachael*

### Managing varying working patterns

The team all had different contracts, working days and work-life patterns. We overcame the challenges this created by working together through constant communication, establishing timelines, and setting deadlines to keep us on track. We found that it was important to give space and time during the meetings to identify problems and come up with solutions.

Each member of the core InCLUDE team has brought their own experience and expertise to the project. However, we were all on contracts with different number of working days. There is a challenging relationship between time contracts and equity of involvement and workload. *Shayma*

I’ve also found it challenging navigating everyone’s different work-life patterns and priorities. I’m learning that early communication and clear deadlines are really helpful for us being able to work together and flexibly—as a last-minute person, this isn’t something that comes naturally to me. *Rachael*

### The emotional commitment

Striving for equality and diversity is an arduous task that requires emotional investment. This is especially the case because progress is gradual and takes time as we work within a landscape of structural and systematic injustice. These challenges are witnessed within our own locality and internationally, and impact on psychological wellbeing. It is therefore especially important to not become disheartened and to celebrate the small achievements that are part of a wider movement for justice and equality.

My lowest point during this research process was when I went [on holiday] to Jerusalem. I felt so upset after witnessing so much injustice…I have learnt that equality and diversity can be achieved but a lot of hard work is required. All good can also be lost very quickly and we must cherish what good we have in our life and work to preserve this and strengthen this before it can be lost. *Sabbir*

We also had to manage feelings of frustration and disappointment in our attempts to overcome some of the barriers to improve recruitment of our underserved groups for the study. We used our team meetings to talk about failed attempts and lack of success, while simultaneously using these feelings and conversations to enhance our learning, improve our thinking, and determination.

Before even delivering the session, recruiting male carers to attend the session was proving difficult in itself, for me, this reinforced the underlying and wider issue of under-representation. Fortunately, a joint effort from all team members allowed us to get sufficient participants to run this group. *Sakab*

Engagement with South Asian carers has been one of our priorities on the IncLUDE project, but it has also been the most challenging. We’ve had a personal connection with a faith-based Independent Fostering Agency which has been helpful. The co-researchers have assisted with recruitment by speaking to their carers directly. *Shayma*

I have been really inspired by the persistence of the team. I can see that this project hasn’t always been easy—it has stretched us emotionally and we have all faced challenges this year, but everyone has stuck together as a team, and I am grateful for the honesty and integrity that everyone brings to the team. *Rachael*

### Creating a safe space

Throughout the project we strived to create a safe space for our team to express and share their thoughts freely and thus create the opportunity to learn and grow, but this was not without its challenges. This included working out how to facilitate groups that included people who have lots of opinions with those who are more reserved.

One of the challenges we have faced within InCLUDE is keeping an inclusive and safe environment within all our research spaces. We have tried our best to welcome everyone who wants to join the InCLUDE project, but this has come with challenges. For example, in an early group, I found it hard to balance hearing out people who had lots of opinions to share with making space for those who were naturally more reserved to join in. Afterwards, the team reflected that having clear guidance around limited time, and telling people that we might move them on, was a helpful way to avoid this. *Rachael*

As a team, we reflected on the first session… to mitigate some of these issues, we decided that we would rectify this by communicating some changes and implementing some ground rules to the group prior to the beginning of the next session. These changes allowed other members of the group to share their experiences and opinions. *Sakab*

### Achieving digital literacy and research skills

The move towards online communications and meetings following the Covid-19 pandemic has become the norm and it is easy to forget that this is not the reality for many people. Both Sabbir and Sakab commented on barriers to online meetings, including the technical skills needed, and difficulties with establishing rapport and reading body language. However, training, problem solving and making some changes helped them to adapt and see the benefits. In academia, there is an assumption that most people are proficient with software such as Excel, Word and so on as they are used daily. However, the importance of IT (information technology) training should not be underestimated for people who do not have these skills.

During my journey to research, I faced different degrees of challenges. I faced some difficulty using the Excel and Microsoft Word to a more advanced level. My colleagues were very accommodating. Rachael helped me better understand Excel and Microsoft word by talking me through the software and sharing online videos. *Sabbir*

Even with the support of my colleague, I found delivering and facilitating the first of the two sessions [of focus groups] quite difficult. This was most probably due to my lack of IT skills and knowledge. Although there are some clear benefits of digitalisation in research such as reach, convenience, efficiency etc., there are also some definite drawbacks such as the need for IT awareness and skills, potential lack of rapport between participants, inability to monitor body language/interest etc. *Sakab*

Sabbir and Sakab commented that taking part in research training strengthened their understanding of research methods, the study, and the importance of ethics. This included Anna Freud qualitative research training completed by Sabbir and Good Clinical Practice completed by both. However, some of the training could have been done earlier in the project.

I feel some the research training for our two co-researchers could have been better planned and delivered from the start. One team member gained a lot of knowledge and skills from the Anna Freud research training, but this was then put on hold while changes were made at the centre, so the other team member did not get the same opportunity. The Good Clinical Practice could also have been done during the induction phase. *Shayma*

Researchers make refences to the need to be reflective; to be mindful of their own thoughts, background and potential bias. This was not always easy as Sakab found that his fostering experiences evoked strong emotions within him. He realised it was important to be aware of these emotions, to acknowledge them so that bias in not introduced into the work. He also acknowledged the challenge of taking on the role of a researcher as a social worker.

On a personal level, I do think it has been advantageous to have experience both as a foster carer as well as a social worker, but at times I have found myself to be unsure as to which hat I should be wearing, I need to be more mindful of this at times, especially when engaging with other professionals and carers. *Sakab*

## Theme five: Reflections on learning

The coproduced journey was also a journey of learning, and the team highlighted how the reflective accounts were used throughout the project to reflect on this learning, leading to the development of this fifth theme.

Learning from InCLUDE was part of an iterative process of continuously reflecting, finding solutions, and making adaptions to achieve the project aims and objectives. We used all the data from the study to improve inclusive recruitment within the Reflective Fostering Study.

The InCLUDE project has come a long way since it first began back in April 2022. We have listened carefully to the feedback from our advisory group and used this knowledge to improve recruitment and made some big changes. The action-research cycles of the project (You said, we did!) have enabled us to rethink strategies, highlight our gains but also be honest about challenges and lack of progress in certain areas. *Shayma*

The diaries elucidate the huge amount of knowledge gained from InCLUDE. This was in the form of content knowledge on foster care, kinship care, mentalisation, discrimination, barriers and facilitators to taking part in research, underrepresentation, diversity and inclusion. This happened in a non-traditional way: the source was not a textbook, research paper or documentary, but by talking with each other, carers and professionals. We increased our knowledge and empathy towards all carers, and in particular kinship carers—Shayma, Sabbir and Sakab had never heard the term ‘kinship carer’ and felt this lack of awareness was reflected in society. Shayma and Rachael were most surprised by the burdens faced by male carers in the female dominated world of foster care.

Learning about the barriers our male carers faced helped me see that anyone can be marginalised, depending on the circumstances, stereotypes, and who is the dominant group. *Rachael*

My task of cleaning the transcript for the ‘kinship carers’ group reinforced my learning from my previous task during kinship carers awareness week. This allowed me to build on my previous understanding and helped me better understand the plight of kinship carers. I soon realised there was differences and points of contention between foster carers and kinship carers. I found that by acknowledging the experiences of such groups, and demonstrating your understanding of these deeper points, will help researchers really connect with the groups they are trying to connect with. *Sakab*

We came away from the project with a rich understanding of the barriers preventing carers taking part in research, such as carers scepticism about research, lack of confidence, experiences of discrimination and marginalisation. Sakab commented that getting people to see research as a vehicle for change is key while also having a streamlined process to improve recruitment.

I am also starting to understand that pessimism is a factor for some people such as when they start to doubt that their contribution will make any difference. I have also realised that some people lack confidence to make contributions. *Sakab*

The importance of building trust within co-production came out strongly in the diaries. Rachael pointed out that context is important. Many carers have no trust because they have been let down by the children’s social care system, people in positions of trust, or have experienced discrimination. There is a strong focus on ethics and safeguarding within research, but the element of trust is one that is missing.

I have been surprised to hear from our advisory groups how many people don’t trust research or researchers. It’s easy to forget when we put so much effort into ethical and safe research that most people don’t see this, or don’t believe it. *Rachael*

The team reflected on the issue of intersectionality and diversity within each underserved group and how this needs to be considered within co-production. There is no single approach that works for all and individual barriers to participation can be unique. However, being inclusive also needs to be balanced with pragmatism. Further attention is needed to include those carers who are particularly marginalised, such as older kinship carers who don’t speak English.

I have realised that there is so much diversity amongst people, even within specific groups and sub-groups. Therefore, I don’t feel there is a single approach to obtaining views and opinions that will tick all boxes, rather, researchers need to adopt the most pragmatic and robust approach that will tick most boxes in order to accommodate as many people as possible, and perhaps have a contingency to pick up those who slip through the net. *Sakab*

Calling participants to conduct the online survey was an opener as I realised this niche group of people had valuable contributions which otherwise would have been missed. *Sabbir*

## Theme six: Personal development

One of the outcomes of the project that had not been anticipated was the development of personal and interpersonal skills among the team; an achievement that extended beyond the project’s stated aims and objectives. This resulted in ‘personal development’ emerging as a theme.

All members spoke about how empowering it was to co-produce the project and how the learning would be taken with them after InCLUDE ended. Sabbir and Sakab came to see that research could make a difference and were inspired to look for further opportunities to contribute to research.

I now see the world differently. I analyse in a different way. I can see so much inequality and the need for diversity and inclusion more now than ever before. We are still very far from our goal of total diversity, freedom and inclusivity. *Sabbir*

Sabbir become more confident to speak up about equality and diversity to audiences. He also became interested in the clinical side of the Reflective Fostering Programme and became a group facilitator. In his diary, he documented some of his learning in relation to meeting formats, working to deadlines, taking minutes and uploading them on the SharePoint for the team. He also picked up skills such as learning how to interject and facilitate focus groups so as to maintain focus on the topic guide and relevant points of discussion.

Coming towards the end of this exciting journey I now feel more confident to speak out about inequality and raise awareness. I am more confident to address an audience and have acquired many new skills that will surely aid me in life moving forward.* Sabbir*

Sakab felt research allowed for critical thinking and innovation. He found that taking minutes of the meetings, gave him a deeper understanding of the subject matter; and this in turn helped him to contribute better. The process of transcribing the focus group recording also aided his understanding about rigorous and thorough research. At a human level, he found himself more empathetic to other carers.

I am beginning to understand the importance of communicating that all views and voices will be heard will be key to improving participation, especially amongst those who are already pessimistic. Participants need to feel valued, and that they can trust you before they fully engage in any research programme. *Sakab*

Another unintended outcome was the positive impact taking part in InCLUDE had on individual carers who were part of the InCLUDE-ME Advisory group or who took part in a focus group. We witnessed compassion, consideration and support being offered between carers during discussions.

I was struck by a South Asian carer who commented that this was the first time she had chance to sit (virtually) with other South Asian carers and discuss the challenges of her fostering journey. Carers enjoyed being in the company of other carers like them. They supported and validated each other. These were nice experiences to witness. *Shayma*

One of the outcomes for which I am most proud is that members of the InCLUDE-ME advisory group were able to help another member access support as a carer for the first time. This can’t be quantified for our funders, or even captured in most research reports, but it is potentially life changing for that carer and I’m so grateful we played a part in facilitating that. *Rachael*

## Reflecting on others’ diaries

We documented the end of our journey through a final discussion on the process of reading and analysing each other’s diaries and our overall experience of the project.

The team enjoyed reading each other’s diaries and found that the style and language used reflected the personalities of each team member. We found the accounts to be powerful, inspiring and showed the passion and sincerity of the team; and everyone considered it a privilege to have embarked on this learning journey together.

Experts by foster caring experience researchers felt heard and supported which was reassuring to the academic researchers. Similarities were noted between Rachael and Shayma on how to balance PPI and research tasks as they reflected on the meaning of authentic co-production, and the challenges in maintaining an inclusive and safe environment. Sabbir and Sakab reflected on how they initially felt underqualified and inexperienced, and the difficulties related to IT and understanding of research processes. All the team talked about the challenges of recruitment, and the need for different approaches to engage with different audiences and research participants.

Overall, I feel that I have come a long way as I am able to grasp information and concepts which were seemingly too complex for me at the beginning of my journey. I struggled so much that at one point, I contemplated leaving my role. It was only the empathy, kindness and accommodating nature of my colleagues which influenced my decision to persevere. *Sakab*

It made me realise how much we have all grown in confidence and knowledge over the last year. It was interesting to see the different angles everyone took and how our personalities shone through. *Rachael*

The discussion on the reflections made me realise how rewarding but also challenging this journey has been for us all; and made me think perhaps we haven’t appreciated how well we have bonded and how far we have come! *Shayma*

This process made me bond better with other members and most of all cascaded the human element. I know my fellow researchers are human just like me, but by reading their diary accounts it really cemented the message that they too have similar thoughts, ideas and have also experienced trials and tribulations. *Sabbir*

## Discussion

Co-production is often spoken about as an approach or methodology, but the InCLUDE project has demonstrated to us that it is primarily about people, who become a team and collaborate to achieve a goal. The team members came into this project with different experiences and motivations, but all with the aim of wanting the work to be credible and have impact. Foster carers as researchers, enriched by their diverse backgrounds, insights, and perspectives, working collaboratively with academic researchers, resulted in the development of a project that was experienced as inclusive, equitable and democratic.

Drawing on all the learning from the themes, the four core researchers, alongside KI and AR-C, developed a series of learning points to share with other researchers, as they embark on their own co-production journeys. These were developed based on what the team felt were the most salient aspects of their own co-production experience as discussed in the themes and are summarised under two headings: supporting co-production in the early stages and overcoming the challenges of co-production.

### Supporting co-production in the early stages:

- All researchers bring different knowledge and experiences; it is the collaboration of alternative perspectives that gives strength to co-production. Experts-by-experience researchers can struggle to appreciate the value of what they bring, so it is important to emphasise that everyone has unique expertise. Academic researchers talking about their own uncertainties and vulnerability, and group discussions about power dynamics, can also facilitate creating a more equitable environment.
- To maintain enthusiasm and facilitate connections among team members, face-to-face meetings should be prioritised over virtual meetings, where possible. Otherwise, aim to have initial meetings face-to-face and use this as an opportunity to get to know each other, rather than discussing the project in detail. Creating a safe space is also crucial and enables the expression and sharing of thoughts freely, thereby creating further opportunity for learning and growth.
- InCLUDE was navigated by harnessing the diversity of the team’s strengths and personalities. Equal does not mean all being the same. Assigning tasks based on interest and developmental needs was key, and this needed to be underpinned by regular discussion of training needs. Some training may appear minor, such as those related to enhancing digital skills, but they are equally as important.

### Overcoming the challenges of co-production:

- All the researchers involved faced challenges during their time in this project, some similar and some different. It is important to recognise the different lifestyles and challenges faced by each member, and structure support and workstyles around them.
- Co-production in research requires a significant investment of time and emotional energy. It is important to acknowledge that it can be an arduous process and it can take time to adapt and get comfortable with a co-production approach. There is a significant amount of learning on the job and vulnerability involved, especially at the start so it is important to regularly check in with each other and work together to keep morale high.
- The use of academic jargon needs to be avoided, and a system established to ensure experts-by-experience researchers can be introduced to the language in a helpful and non-intimidating manner.
- Develop a working routine that works for all members of the team; for us this included a supportive environment, good communication, flexibility, clear and agreed deadlines, and a recognition that all ideas are important and that all opinions are valuable.
- In this project, experts-by-experience researchers working one day a week was a pragmatic decision, working in the project alongside their fostering role. Ensuring equity of involvement, including in decision making should therefore be managed carefully, to ensure everyone has a sense of ownership, regardless of their working hours.

## Strengths and limitations of reflective accounts

The use of diaries enabled us to document our unique experiences throughout our journey and capture how we made sense of the project at an individual and collective level. The diaries allowed capturing and reflections on learning which could easily be missed or forgotten once the challenges had passed. By recording thoughts, feelings, and experiences in real-time, we became more self-aware. Looking through the diaries at the end of the project, we could see progress, achievements, empathy, mutual understanding, and the importance and impact of seeing an issue from a different perspective.

However, with busy schedules diaries tended to be used on an ad hoc basis. Moving forward, we would encourage researchers to keep track of their learning though diaries, but to be consistent in their use, and to share and discuss them regularly within a safe space. Some may be more comfortable with creating voice notes or capturing their experiences in other formats. We would also suggest explicit references to researcher reflectivity; on reflection we feel this was not sufficiently captured in our own diaries.

This paper draws on reflective accounts that were a collation of individual diary entries curated by the author of each diary—this included both raw diary text but also additional reflections. We felt this approach worked well for our team as it allowed each researcher to reflect on what they wrote, remove anything which felt too personal or confidential to share, and to help entries make more sense to readers. However, it does create a further level of remove from the actual day-to-day realities, as in the reflective accounts we were aware of writing for an external audience.

The reflective accounts were independently read by the four researchers, a project advisor and external researcher to reduce the chance of bias being introduced when it came to selection of themes. The paper was also written collaboratively and shared at each stage to ensure everyone had the chance to provide further feedback and discussion.

## Conclusion

This paper was constructed as a response to the need for more reporting on the processes and lived experience of co-production [45/46]. It provides a novel and unique contribution to the literature by documenting the lived experiences of co-production within the context of foster care. The use of reflective accounts captured the story of the InCLUDE project process, and the experiences of academics and foster carers working together collaboratively as equal partners in the research. Of particular interest is how much the experiences/feelings of the researchers mirror each other, regardless of the previous experience of conducting research. It may therefore be of value for other researchers to know that anxieties about embarking on a co-production research project are likely shared among team members, and that creating open space to discuss these early on, such as building reflection time into team meetings, is likely to be helpful.

While practice and policy developments often prioritise measurable outputs, this paper underscores how subjective learning and attitudinal shifts are equally vital outcomes in the development of impactful research and co-production. Among our team, these were some of the most significant personal achievements, but these are not traditionally captured, which means that opportunities for the research community to learn about subjective impact is often lost. The reporting of the less tangible, personal outcomes of projects, as well as research outcomes, provides a richer narrative, broadens our understanding of impact, and can help researchers plan research in a holistic way.

In conclusion, while there is an increasing emphasis on addressing issues of inclusion and representation in research, and the importance of collaboration with ‘experts-by-experience’, the guidance does not often capture the ‘lived experience’ of working this way, from the perspective of all those involved. By sharing our learning of co-production, we hope to inform others undertaking similar work, helping them to do it more effectively. We also hope to have demonstrated how the use of reflective accounts can be used by researchers as an opportunity to become more familiar with both the processes involved in co-production, and work trying to improve representation and diversity in research.

## Supplementary information

Additional file1

## Data Availability

The datasets generated and/or analysed during the current study are not publicly available due to the inclusion of sensitive information but are available from the corresponding author on reasonable request.

## Abbreviations

NIHR: National institute for health research
RFS: Reflective fostering study
InCLUDE: Increasing collaboration and learning with underserved communities for diversity and equity
EDI: Equality, diversity and inclusion
PPI: Patient and public involvement
IT: Information technology
