# Supplementary material for: The lived experience of co-production: Reflective accounts from the InCLUDE project
Source: Res Involv Engagem. 2024 Oct 14;10:104. doi: 10.1186/s40900-024-00639-2 (PMC11475932; doi:10.1186/s40900-024-00639-2)
Supplement: Supplementary file 1 — Additional file1 [file 40900_2024_639_MOESM1_ESM.docx]

| **Section and topic** | **Item** | **Reported on page No** |
| --- | --- | --- |
| 1: Aim | This is a co-produced paper about the experience of four researchers (two experts by foster caring experience and two experts by profession) two research experts by profession involved in a co-produced project called InCLUDE. The project explored the barriers and facilitators to inclusive research in foster care, and aimed to improve representation of kinship foster carers, male carers and South Asian carers in a large, randomised control trial called the Reflective Fostering Study. | 4,6,7 |
| 2: Methods | The four researchers kept a weekly reflective diary of their experiences and learning from the InCLUDE project. At the end of the project, each researcher collated their diary entries into a reflective account of their journey within the InCLUDE project. The reflective accounts were then shared with each other, and two other researchers. All six researchers independently read all the reflective accounts and identified common themes, and similarities and differences. They then came together and discussed common themes and narratives across the accounts which would be most helpful for other researchers to learn from. | 7,8,9,27,28 |
| 3: Study results | This paper highlights that, in addition to positive changes to promoting inclusivity within foster care research, the lived experience researchers gained knowledge, skills, confidence and new friendships throughout the project. The paper also discusses the challenges faced over the duration of the InCLUDE project, by each researcher. | 9,10,11,12,13,14,15  16,17,18,19,20,21,22  23,24,25,26 |
| 4: Discussion and conclusions | InCLUDE was centred around a coproduction approach within all our activities and planning. The lessons from engagement in this process put forwards by the four researchers are summarised in the discussion section of the paper. | 28,29,30,31 |
| 5: Reflections/critical perspective | The diversity among the coproduction team contributed to the project success. The varied genders, ethnicities, caring experience, and previous research experience meant there were varying perspectives and opportunity for critical reflection. In particular, the two team members with foster carer experience brought unique insights and personal access to knowledge and spaces which the professional researchers could not have.  The paper reflects that each researcher found the initial establishment of the coproduction team challenging and questioned their expertise and value. Through honest conversations and with time, a strong team dynamic and trusting relationships developed.  Some of the reflections in this paper may have been edited by the researchers, as they knew entries would be read by others. | 28,29,30,31 |
